# Eltrombopag can promote platelet implantation after allogeneic hematopoietic stem cell transplantation as safely and similarly to thrombopoietin

**DOI:** 10.3389/fimmu.2024.1340908

**Published:** 2024-04-08

**Authors:** Yahan Li, Fansheng Kong, Guanchen Bai, Yujie Jiang, Wenlu Zhang, Xue Sun, Xiaohui Sui, Ying Li, Mei Ding, Dai Yuan, Xin Wang, Xiaosheng Fang

**Affiliations:** ^1^ Department of Hematology, Shandong Provincial Hospital Affiliated to Shandong First Medical University, Jinan, Shandong, China; ^2^ Department of Hematology, The Affiliated Hospital of Shandong University of Traditional Chinese Medical, Jinan, Shandong, China; ^3^ Department of Hematology, The Affiliated Taian City Centeral Hospital of Qingdao University, Taian, Shandong, China; ^4^ Department of Hematology, Shandong Provincial Hospital, Shandong University, Jinan, Shandong, China; ^5^ Branch of National Clinical Research Center for Hematologic Diseases, Jinan, Shandong, China; ^6^ National Clinical Research Center for Hematologic Diseases, the First Affiliated Hospital of Soochow University, Suzhou, China

**Keywords:** eltrombopag, thrombopoietin, safety, prognosis, allogeneic hematopoietic stem cell transplantation, platelet engraftment

## Abstract

**Background:**

Eltrombopag has demonstrated efficacy in treating low platelet (PLT) levels, but it remains unclear whether eltrombopag can promote PLT engraftment after hematopoietic stem cell transplantation (HSCT).

**Methods:**

Forty-one HSCT patients received eltrombopag 50 mg/d from +1 day until PLT >50 × 10^9^/L or 1 month after HSCT. Fifty-one patients in the same period received thrombopoietin (TPO) to promote PLT graft after HSCT and served as a control group.

**Results:**

A total of 51 patients who applied TPO during the same period were treated as a control. In the eltrombopag group, the median time to white blood cells (WBC) graft was 12 days (range, 10-17 days) and the PLT graft was 15 days (range, 10-30 days), whereas for the patients in the TPO group, the median time to WBC and PLT graft was 12 days (range, 9-23 days) and 15.5 days (range, 9-41 days), respectively. In the first month after HSCT, the median WBC count in the eltrombopag group was 4.41 × 10^9^/L (range, 0.87-40.01 × 10^9^/L) and the median PLT was 89x10^9^/L (range, 30-401 × 10^9^/L); the median WBC and PLT \counts in the TPO group were 4.65 × 10^9^/L (range, 0.99-23.63 × 10^9^/L) and 86 × 10^9^/L (range, 5-512 × 10^9^/L), respectively. Patients in the TPO or eltrombopag group did not experience serious side effects after drug administration, and the difference in side effects on liver and kidney function between the two groups was not statistically significant.

**Conclusion:**

Eltrombopag is safe and similarly promotes platelet engraftment to thrombopoietin after allogeneic HSCT.

## 1 Introduction

Hematopoietic stem cell transplantation (HSCT) is an effective curative measure for many hematologic malignancies and some nonmalignant diseases. Neutrophil and platelet (PLT) implantation after HSCT is essential for optimal results (1). Prolonged isolated thrombocytopenia (PT) is a very common complication for all patients with HSCT. PT patients are usually faced with uniformly poor outcomes (2, 3). Thus, how to reduce the incidence of PT and successfully manage thrombocytopenia after HSCT remains a major challenge.

Thrombopoietin (TPO) is a cytokine, and previous studies have shown that TPO is the main physiological cytokine that stimulates platelet production (4, 5). A prospective randomized controlled study has shown that recombinant human TPO (rhTPO) promotes platelet engraftment in patients after HSCT (6). The safety of rhTPO has been demonstrated in many previous trials (6, 7).

Eltrombopag is a small molecule non-peptide oral formulation that is an agonist of the thrombopoietin receptor, and it can increase PLT counts in patients with thrombocytopenia. Although initially used to improve thrombocytopenia in chronic immune thrombocytopenia (ITP), it was later found to be effective in other causes of thrombocytopenia (8). Since thrombopoietin receptors are expressed on both megakaryocytes and hematopoietic stem cells, hematopoietic stem cell can be stimulated by eltrombopag (9–11). More recently, eltrombopag has been used for the treatment of thrombocytopenia and graft failure after HSCT (12).

The effects of eltrombopag on poor graft function of HSCT patients have been studied and were shown to be effective (13, 14). However, it remains unclear whether eltrombopag can promote PLT engraftment after HSCT.

Herein, we conducted a retrospective, single-arm clinical trial to evaluate the effect of eltrombopag on platelet engraftment in patients after transplantation and the safety of clinical use of eltrombopag.

## 2 Materials and methods

### 2.1 Study design

We enrolled a total of 94 patients diagnosed with hematological malignancies who underwent HSCT from three hospitals from 21 January 2019 to 10 November 2021. The three hospitals both used TPO or eltrombopag after transplantation as standard treatment. These patients were divided into two groups. Patients were administered 50 mg (qd) from +1 day until PLT >50 × 10^9^/L or 1 month after HSCT were classified as the eltrombopag group, and patients who used TPO only during the same period were divided into the TPO Group. We verified ANC and PLT counts daily for post-transplant patients. Additionally, we monitored kidney and liver function every three days. We compared the implantation of white blood cells (WBC) and PLT of the two groups after HSCT. We also analyzed the side effects of both drugs on patients’ liver function, kidney injury, and outcomes. This study was approved by the institutional ethics board of Shandong Provincial Hospital Affiliated to Shandong First Medical University, the Affiliated Hospital of Shandong University of Traditional Chinese Medicine and Taian City Central Hospital. This study was performed in accordance with the Helsinki Declaration of 1975 and was approved by the institutional ethics board of Shandong Provincial Hospital (Jinan, China), the Affiliated Hospital of Shandong University of Traditional Chinese Medicine (Jinan, China), the Affiliated Taian City Center Hospital of Qingdao University (Taian, China). Informed consent.

### 2.2 Conditioning regimen and prophylaxis of GVHD

All patients received myeloablative conditioning regimens including busulfan + cyclophosphamide (busulfan 0.8 mg/kg in 9 doses, cyclophosphamide 50 g/kg/day, days -3, -2), busulfan + fludarabine (busulfan 0.8 mg/kg in 9 doses, fludarabine 30mg/kg/day, from day -6 to day-2),or total body irradiation + cyclophosphamide (total body irradiation 3Gy/day, from day -6 to day-4, cyclophosphamide 50 g/kg/day, days -3, -2)-based regimens. To prevent GVHD, cyclosporine A, mycophenolate, and short-term methotrexate were administered in all patients. Patients who underwent matched HSCT from sibling donors did not receive ATG, and the other patients received ATG (2.5 mg/kg/day, from day -5 to day -2).

### 2.3 Stem cell mobilization and infusion

Granulocyte colony-stimulating factor (5-10 μg/kg/day) was used to mobilize hematopoietic stem cells into the peripheral blood. Peripheral blood stem cells were collected on the fifth day after mobilization. The ideal infused mononuclear cells were more than 5 × 10^8^/kg, and CD34^+^ cells should were more than 2 × 10^6^/kg.

### 2.4 Indicators and definitions

WBC engraftment after HSCT was defined as neutrophil granulocyte count greater than 0.5 × 10^9^/L for three consecutive days. PLT engraftment was defined as a PLT count exceeding 20 × 10^9^/L for three consecutive days without transfusion support. We also compared the time achieved for the WBC count>0.2 after HSCT and the number of WBC and PLT at 1 and 3 months after HSCT between the two groups. Liver function was assessed by analyzing the levels of glutamic oxaloacetic transaminase (AST), glutamic-pyruvic transaminase(ALT), alkaline phosphatase (ALP) and total bilirubin (TBIL). Kidney injury was assessed by measuring urinary protein levels (Upro), blood urea nitrogen (BUN), and creatinine (CREA). The grade of liver and kidney injury was determined according to the World Health Organization (WHO) classification system. Parallelly, we collected the number of MKCs in the bone marrow of patients one month after HSCT. Acute GVHD (aGVHD) was accessed according to the Glucksberg criteria (15) and cGvHD was graded based on the revised Seattle criteria (16). Overall survival (OS) was defined as the time between HSCT and death. Progression-free survival (PFS) was defined as the time between HSCT and disease recurrence or death.

### 2.5 Statistical analysis

The T test was used to compare continuous variables and the chi-square test was used to compare categorical variables. The Kaplan-Meier method and Cox proportional hazard model were used to estimate leukocyte and PLT engraftment, OS, PFS, and GVHD. SPSS v.25.0 was used for data analysis. P<0.05 based on the 2-sided hypothesis tests were considered statistically significant.

## 3 Results

### 3.1 Patients’ characteristics

This study involved 94 consecutive patients. A total of 43 patients received eltrombopag after HSCT, among whom 2 individuals experienced severe nausea and vomiting, making oral medication intolerable. Subsequently, they switched to treatment with thrombopoietin (TPO). As these two patients underwent cross-over treatment with two different medications, they were not included in a specific treatment group for analysis. A total of 41 patients finally enrolled in the eltrombopag group. A total of 51 patients used TPO and were enrolled in the TPO group. The patient characteristics are summarized in **Table 1**. There was a significant difference in follow-up time between the eltrombopag group and the TPO group. There were no significant differences in sex, recipient’s sex, patient’s age, pre-HSCT CR, donor-recipient HLA and other indexes between the two groups (**Table 1**).

**Table 1:** Characteristics of the patients.

Characteristics	Eltrombopag group (n=41)	TPO group (n=51)	P-value
Doners’ gender, N(%)			.531
Male	24 (59)	26 (51)	
Female	17 (41)	25 (49)	
Recipient’s gender Male Female	27 (66) 14 (34)	37 (73) 14 (27)	.504
Doners’ age (median, range), years Recipient’s age (median, range), years Diagnosis, N(%) AML ALL MDS Others Pre-HSCT CR, N(%) CR1 ≥CR2	31 (10-65) 33 (11-54) 20 (49) 12 (29) 6 (15) 3 (7) 28 (68) 13 (32)	29 (8-64) 32 (8-58) 25 (49) 14 (27) 4 (8) 8 (16) 34 (67) 17 (33)	.196 .592 .519 > .999
Donor-recipient HLA matching, N(%) 10/10 matched 9/10 matched 7/10 matched 6/10 matched 5/10 matched Donor-recipient sex matching, N(%) F-F F-M M-F M-M Donor-recipient ABO matching, N(%) Matched Major mismatch Minor mismatch Bidirectional mismatch Graft Source, N(%) PB PB+BM	10 (25) 1 (2) 1 (2) 6 (15) 23 (56) 5 (12) 9 (22) 12 (29) 15 (37) 21 (51) 8 (20) 9 (22) 3 (7) 35 (85) 6 (15)	14 (27) 2 (4) 2 (4) 7 (14) 26 (51) 5 (10) 9 (18) 20 (39) 17 (33) 31 (61) 10 (20) 7 (13) 3 (6) 44 (86) 7 (14)	.992 .829 .733 > .999
Infused cells, median (range)			
MNC, ×10^8^/kg	8.90 (4.40-26.36)	8.76 (2.50-22.57)	.217
CD34^+^ cells, ×10^6^/kg Time from diagnosis to HSCT (median, range), months Follow-up time (median, range), months	3.76 (1.38-15.04) 5.07 (0.63-95.67) 9.50 (1.03-19.23)	3.81 (0.61-12.04) 5.10 (0.53-63.63) 18.90 (2.37-27)	.491 .720 < .001

TPO, thrombopoietin; AML, acute myeloid leukemia; ALL, acute lymphoblastic leukemia; MDS, myelodysplastic syndromes; HSCT, hematopoietic stem cell transplantation; CR, complete response; HLA, human leukocyte antigen; PB, peripheral blood; BM, bone marrow; MNC, mononuclear cell

### 3.2 Engraftment

**Figure 1:**
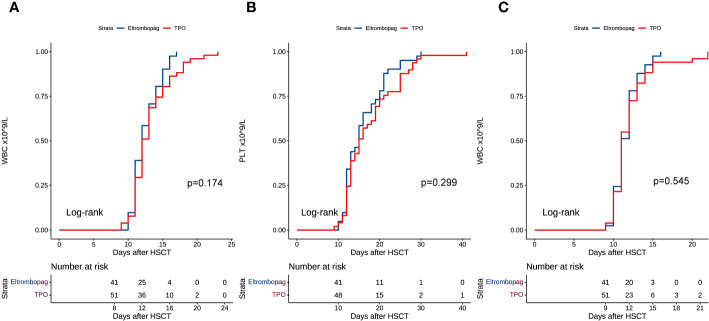
**(A)** WBC engraftment after HSCT. **(B)** PLT engraftment after HSCT. **(C)** Time course of WBC counts >0.2 × 10^9^/L after HSCT.

All patients achieved engraftment, and none displayed primary graft rejection. The median time to recovery from WBC was 12 days (range, 10-17 days) in the eltrombopag group and 12 days (range, 9-23 days) in the TPO group (P = 0.174, HR = 1.344, 95%CI: 0.877-2.059) (**Figure 1A**). Two patients had poor engraftment of PLT in the TPO group. The median time to PLT engraftment was 15 days (range, 10-30 days) in the eltrombopag and 15.5 days (range, 9-41 days) in the TPO group (P = 0.299, HR = 1.249, 95%CI: 0.821-1.901) (**Figure 1B**).

We compared the time to WBC counts >0.2 × 10^9^/L in both groups. In the eltrombopag and TPO groups, the median time to WBC >0.2 × 10^9^/L was 11 days (range, 9-16 days) and 11 days (range, 9-22 days), respectively (eltrombopag vs TPO: P = 0.545, HR = 1.138, 95%CI: 0.749-1.729) (**Figure 1C**). We collected the = WBC and PLT counts at 1 and 3 months after HSCT. At the first month after HSCT, the median WBC count was 4.41 × 10^9^/L (range, 0.87-40.01 × 10^9^/L) in the eltrombopag group and 4.65 × 10^9^/L (range, 0.99-23.63 × 10^9^/L) in the TPO group, respectively (P = 0.720, HR = 1.079, 95%CI: 0.711-1.637), and 3.96 × 10^9^/L (range, 2.19-21.69 × 10^9^/L) and 4.21 × 10^9^/L (range, 1.59-10.41 × 10^9^/L) at the third month after HSCT, respectively (P = 0.371, HR = 0.819, 95%CI: 0.529-1.268). While for PLT it was 89 × 10^9^/L (range, 30-401 × 10^9^/L) and 86 × 10^9^/L (range, 5-512 × 10^9^/L) in the TPO and eltrombopag group at the 1st month, respectively (eltrombopag vs. TPO: P = 0.198, HR = 0.761, 95%CI: 0.503-1153). The median PLT count was 91 × 10^9^/L (range, 3-299 × 10^9^/L) and 90 × 10^9^/L (range, 9-261 × 10^9^/L) in the two groups at the third month, respectively (Eltrombopag vs TPO: P = 0.625, HR = 0.897, 95%CI: 0.581-1.387).

One month after HSCT, all patients in the eltrombopag group had PLT >25 × 10^9^/L, while in the TPO group 89% of the patients had PLT >25. Overall, 89% of the patients in the eltrombopag group had PLT >25 × 10^9^/L, while in the TPO group, 89% patients had PLT >25 × 10^9^/L. Approximately, 89% of patients in the eltrombopag group had PLT >50 × 10^9^/L, while 58% patients in the TPO group had PLT >50 × 10^9^/L. In the eltrombopag group, the PLT counts of 51% patients were over 100 × 10^9^/L, and in the TPO group 42% patients had counts greater than 100. Three months after HSCT, in the eltrombopag group, 90% patients had PLT >25 × 10^9^/L, compared with 89% patients in the TPO group, whereas 77% of the patients in the eltrombopag group had PLT >50 × 10^9^/L, and 71% of patients in the TPO group had PLT >50 × 10^9^/L. There were 52% patients in the eltrombopag group with PLT >100 × 10^9^/L, whereas 49% patients of the TPO group.

The MKCs in the bone marrow were assayed 1 month after HSCT. No MKCs were found in the bone marrow of 3 patients in the Eltrombopag group and 3 patients in the TPO group. The median level of MKC was 44 (range, 2-345) and 58 (range, 2-788) in the eltrombopag and TPO group, respectively (P = 0.425).

### 3.3 Graft-versus-host disease

**Figure 2:**
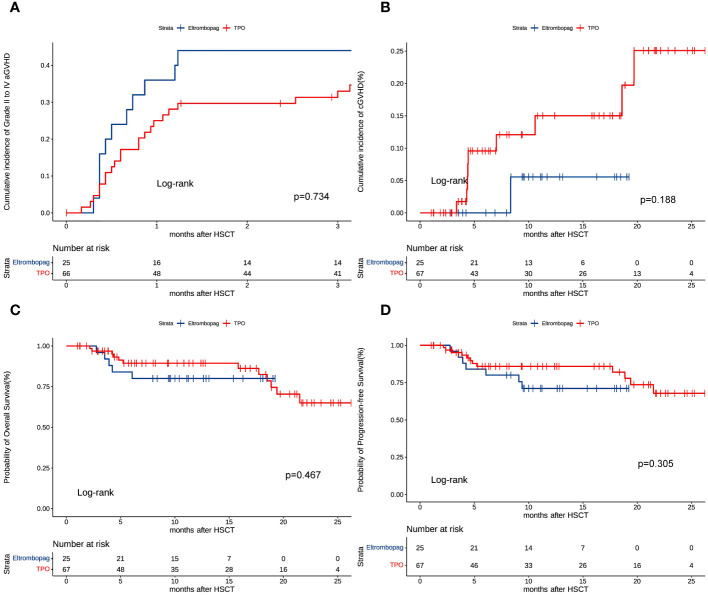
**(A)** Cumulative incidence of grade II to IV aGVHD. **(B)** Cumulative incidence of grade II to IV cGVHD. **(C)** Overall survival (OS). **(D)** Progressive-free survival (PFS).

Sixteen patients developed grade II-IV aGVHD in the eltrombopag group, while 17 developed grade II-IV aGVHD in the TPO group. At 100 days after HSCT, no significant differences were observed in cumulative aGVHD in the eltrombopag (39%, 95% CI: 23%-54%) vs TPO groups (33.3%, 95% CI: 19%-46%) (P = 0.734, HR = 1.125, 95%CI: 0.569-2.228) (**Figure 2A**). One (2%, 95% CI: 1%-7%) patient developed grade II-IV cGVHD in the eltrombopag group and 9 (17%, 95% CI: 6%-28%) developed grade II-IV cGVHD in the TPO group. The number of patients developing cGVHD in the Eltrombopag and TPO group was not significantly different (P = 0.188, HR = 0.246, 95%CI: 0.030-1.988) (**Figure 2B**).

### 3.4 Survival

Survival analysis revealed no statistical difference (eltrombopag vs. TPO: P = 0.467, HR = 1.490, 95%CI: 0.509-4.364) in the 2-year OS in the eltrombopag (85%, 95% CI 74%-96%) vs TPO groups (78%, 95% CI 66%-90%) (**Figure 2C**). The two-year PFS was similar in the eltrombopag group and in the TPO group (80%, 95CI: 67%-93% vs 78%, 95CI: 66%-90%, P = 0.305, HR = 1.664, 95%CI: 0.629-4.403, **Figure 2D**). Six (17%) and 11 (24%) patients died in the Eltrombopag and TPO group, respectively (P = 0.394).

We also compared the adverse effects on the liver and kidney in the eltrombopag and TPO groups after HSCT. We evaluated liver and kidney function throughout the treatment period after transplantation. Based on TBIL levels, 37 patients in the eltrombopag group were assessed as having grade 0 liver injury, 4 patients had grade I liver injury. Forty-two patients were evaluated as having grade 0 liver injury and 7 patients had grade I liver injury in the TPO group, respectively (P = 0.514). According to ALP levels, all patients in the eltrombopag group had a grade 0 liver injury, 44 patients had a grade 0 liver injury, and 5 patients had a grade I liver injury in the TPO group, respectively (P = 0.060). Based on BUN levels, there were 39 patients with grade 0 kidney injury and 2 patients with grade I injury in the eltrombopag group, whereas 45 patients with grade 0 kidney injury and 4 patients with grade I in the TPO group TPO (P = 0.685). Based on CREA levels, all patients had grade 0 kidney injury in the eltrombopag group, 48 of the patients had grade 0 kidney injury, and I patient had grade 1 in the TPO group (P > 0.999). Depending on Upro levels, 24 of the patients had grade 0 kidney injury and 17 patients had grade I in the eltrombopag group. Twenty-four patients had grade 0 kidney injury and 25 patients had grade I in the TPO group TPO (P = 0.365).

## 4 Discussion

HSCT is the only curable treatment for many hematological malignant diseases and some non-malignant diseases, but according to the literature, approximately 5%-44% of patients will experience thrombocytopenia after HSCT (17–19). Long-term thrombocytopenia after transplantation is also a risk factor for a poor prognosis (20). Currently, there is no standard treatment for thrombocytopenia after transplantation. Gamma globulin, androgen, and recombinant human thrombocytopenia (rhTPO) are commonly used in clinical treatment of thrombocytopenia after transplantation. Eltrombopag is an oral small molecule agonist for the thrombopoietin receptor. As early as 2008, eltrombopag was approved by the US FDA for the treatment of primary immune thrombocytopenia (ITP) (21), and effective rates were reported to be 59% to 85% (22). Eltrombopag has been shown to have promising results in patients with ITP and refractory severe aplastic anemia (rSAA), with almost 80% of patients with ITP and 40% of rSAA showing platelet recovery after treatment (23–25). In addition, eltrombopag has recently been used for the treatment of thrombocytopenia after HSCT and showed high effectiveness (13, 26–30). However, it is not clear whether Eltrombopag can promote platelet implantation after HSCT.

TPO is a key regulator of thrombogenesis, which promotes proliferation and multiploidy of megakaryocytes by stimulating stem cells to differentiate into megakaryocytes, thus promoting thrombogenesis (5, 31, 32). Sun et al. (33) treated 24 patients with chronic isolated thrombocytopenia (PT) after HSCT with rhTPO, and the study showed that 45.8% of the patients responded to rhTPO treatment and eventually achieved platelet implantation. Delayed platelet implantation (DPE) is also a common complication after allo-HSCT, Kanda (34) showed that 5-37% of patients who received allo-HSCT developed DPE (19), while Wang et al. (35) demonstrated that rhTPOT could promote platelet transplantation after HSC. IFN-γ, a key pro-inflammatory cytokine, was reported to be involved in the destruction of HSPC by inhibiting TPO signaling, while eltrombopag can bypass this inhibition *in vitro* by activating c-MPL signaling (36, 37). Since the thrombopoietin receptor is expressed in both megakaryocytes and hematopoietic stem cells, eltrombopag can promote the formation and maturation of megakaryocytes to release platelets and promote stem cell generation (38, 39). It has also been reported that not only PLT, but also hemoglobin and WBCs increased significantly with eltrombopag treatment after allogeneic HSCT (25, 40, 41). These studies suggest that eltrombopag may play a better role in promoting hematopoietic stem cell graft.

In our study, there were 2 cases of poor platelet implantation after transplantation among patients with TPO. Except for these 2 cases of poor platelet implantation, the remaining 51 patients in the TPO group and the 41 patients in the Eltrombopag group were successfully implanted with white blood cells and platelets after transplantation. The median leucocyte and platelet grafting time after transplantation were the same in both groups. Furthermore, we found that the patients with the highest leukocyte or platelet implantation after transplantation were both in the TPO group. We also analyzed the number of WBCs and PLTs in both groups at 1 and 3 months after transplantation. WBCs were higher in the TPO group at 1 and 3 months after transplantation, whereas PLT counts were higher in the eltrombopag group at 1 month after transplantation. The number of MKC in the bone marrow 1 month after transplantation was similar between the two groups and there are no statistical differences. From our study, it can be seen that eltrombopag can promote PLT implantation after HSCT, which is the same as confirms findings from a previous study that found that eltrombopag could be used to treat graft dysfunction after transplantation (42, 43).

Early treatment with TPO and Eltrombopag after HSCT had tolerable side effects and high safety (44–47). Han et al. (6) conducted a study including 120 patients with HSCT and found that there was no difference in adverse events involving liver function, kidney function, coagulation function and GVHD between rhTPO group and the control group, and the probability of OS was similar. Another study involved 38 patients who received eltrombopag for thrombocytopenia after HSCT and found that all patients were well tolerated. Twenty-three patients developed aGVHD, but all of these patients recovered without discontinuing eltrombopag. Other serious adverse reactions such as severe liver injury and thrombosis were not observed (48). In our study, only two patients who used TPO developed III-IV aGVHD, none of the patients in the two groups had grade III-IV aGVHD, the liver injury and kidney injury were mild, and none of the patients had other serious adverse reactions. Additionally, patients treated with eltrombopag and TPO had similar OS and PFS. Our study showed the tolerance and safety of TPO and eltrombopag was in accordance with other previously published data (7, 49).

It should be noted that due to the retrospective nature of this study, we cannot determine to what extent platelet recovery after transplantation is affected by TPO or Eltrombopag. Furthermore, this study is limited by the small sample size of patients, which can cause data bias. More prospective randomized controlled large-sample clinical studies are needed to confirm the exact efficacy of early application of Eltrombopag after transplantation.

However, this study has confirmed that eltrombopag can be used to promote platelet implantation in patients early after transplantation, its efficacy is not inferior to that of TPO in promoting platelet implantation, and its side effects can be tolerated and are safe.

## Data availability statement

The original contributions presented in the study are included in the article/Supplementary Material. Further inquiries can be directed to the corresponding authors.

## Ethics statement

The studies involving humans were approved by the institutional ethic board of Shandong Provincial Hospital Affiliated to Shandong First Medical University, the Affiliated Hospital of Shandong University of TCM and Taian City Central Hospital and followed the Helsinki Declaration of 1975. The studies were conducted in accordance with the local legislation and institutional requirements. Written informed consent for participation was not required from the participants or the participants’ legal guardians/next of kin in accordance with the national legislation and institutional requirements.

## Author contributions

YL: Data curation, Software, Writing – original draft. FK: Supervision, Writing – review & editing. GB: Supervision, Writing – review & editing. YJ: Data curation, Methodology, Writing – review & editing. WZ: Software, Writing – review & editing. XS: Methodology, Writing – review & editing. XHS: Methodology, Writing – review & editing. YL: Resources, Writing – review & editing. MD: Resources, Writing – review & editing. DY: Software, Writing – review & editing. XW: Supervision, Writing – review & editing. XF: Supervision, Writing – review & editing.
